# Single Beds

**Published:** 1892-11

**Authors:** 


### SINGLE BEDS.

If these were more numerous than they are, a great many people would be better off. When one is tired, sick, cross, restless, out-of-sorts, he or she ought to sleep alone and not communicate by proximity the maladies that affect him. The brute creatures when sick go away by themselves till they die or get over their troubles, and this instinct a great many human beings have; those that have it are best if indulged in it, not to the slightest degree of neglect, however. Left to themselves, they can compose their internal dissensions, recover their lost equilibrium, and get back their habitual rate of "vibration;" whereas, if continually disturbed, and "crossed," and interrupted, they are a long time in getting back to the normal.

Where two children in a family must share the same room, in a great many cases they would be better off to have two single beds rather than one wide double bed. We can share a great many things with those we love, but solitude clings to us from birth to death. We come into the world alone, we must go out of it alone, and we live in it alone, in a certain important sense, and to get and keep our "bearings," we must sometimes be left alone. It is good that we should be. He who has his bed to himself may be essentially alone for a portion of the twenty-four hours, may have himself to himself, and adjust his internal mechanism to his own satisfaction. For a great many woes and ills, solitude is a balm—what we call solitude—for when alone the immaterial asserts itself, the actual fades, the real is present with us.

